# Biocontrol agent *Fusarium oxysporum* f.sp. *strigae* has no adverse effect on indigenous total fungal communities and specific AMF taxa in contrasting maize rhizospheres

**DOI:** 10.1016/j.funeco.2016.05.007

**Published:** 2016-10

**Authors:** Judith Zimmermann, Mary K. Musyoki, Georg Cadisch, Frank Rasche

**Affiliations:** Institute of Agricultural Sciences in the Tropics (Hans-Ruthenberg-Institute), University of Hohenheim, Stuttgart, Germany

**Keywords:** Biological control agent, *Fusarium oxysporum* f.sp. *strigae*, *Striga hermonthica*, Non-target rhizosphere fungi, Arbuscular mycorrhizal fungi, Environmental safety

## Abstract

We studied the effects of *Fusarium oxysporum* f.sp. *strigae* (Fos), a soil-borne biocontrol agent (BCA) against *Striga hermonthica*, on total fungal and arbuscular mycorrhizal fungal (AMF) taxa in rhizospheres of maize in both clayey and sandy soil. Effects of Fos-BCA ‘Foxy-2’ were evaluated against (1) *S. hermonthica* presence, and (2) organic fertilization with *Tithonia diversifolia* residues at 14, 28 and 42 d after ‘Foxy-2’ inoculation, via DNA-based quantitative PCR and TRFLP fingerprinting. In both soils, ‘Foxy-2’ occasionally promoted total fungal abundance, while the community composition was mainly altered by *T. diversifolia* and *S. hermonthica*. Notably, ‘Foxy-2’ stimulated AMF *Gigaspora margarita* abundance, while *G. margarita* was suppressed by *S. hermonthica*. Total fungal and AMF abundance were promoted by *T. diversifolia* residues. In conclusion, ‘Foxy-2’ resulted in no adverse effects on indigenous rhizosphere fungal communities substantiating its environmental safety as BCA against *S. hermonthica*.

## Highlights

• ‘Foxy-2’ promoted AMF *Gigaspora margarita* abundance.
• Total fungal abundance was only transiently stimulated by ‘Foxy-2’.
• Contrastingly, *G. margarita* abundance was suppressed by *Striga hermonthica*.
• ‘Foxy-2’ induced only a minor alteration of fungal community composition.
• ‘Foxy-2’ effects were generally superimposed by organic fertilization.

## 1 Introduction

The parasitic weed *Striga hermonthica* is a major constraint to cereal production in Sub-Saharan Africa causing yield losses worth US$ 9 billion (Gibbon et al., 2007, Ejeta, 2007). *S. hermonthica* parasitizes staples such as millet (*Pennisetum americanum*), sorghum (*Sorghum bicolor*), maize (*Zea mays*), and rice (*Oryza sativa*) (Elzein and Kroschel, 2004; Marley et al., 2004). It infests more than 50 million hectares of farmland with intensifying dissemination in Sub-Saharan Africa, which makes it one of the gravest threats to food security in this region (Parker, 2012).

Control of *S. hermonthica* remains challenging due to its very high seed production per plant, with seed survival rates in soils of more than ten years (Parker and Riches, 1993, Van Mourik, 2007). It has been widely accepted that a single control method is not effective against *S. hermonthica*, hence, integrated approaches are postulated as control strategies (Menkir and Kling, 2007, Hearne, 2009, Atera et al., 2012).

The combination of biological control agents (BCAs) such as *Fusarium oxysporum* f.sp. *strigae* (Fos) along with tolerant crop varieties provided respectable control against *S. hermonthica* under field conditions in Burkina Faso, Benin and Nigeria (Schaub et al., 2006, Venne et al., 2009). In particular, the Fos strain ‘Foxy-2’ was effective in suppressing all developmental stages of *S. hermonthica* ranging from germination to flowering (Elzein and Kroschel, 2004, Ndambi et al., 2011). In addition, Ndambi et al. (2011) reported that ‘Foxy-2’ colonized endophytically the roots of the host crop (e.g., sorghum), where the biocontrol activity of ‘Foxy-2’ was initialized after *S. hermonthica* attacked the root system.

In contrast to previous studies performed in West Africa (e.g., Schaub et al., 2006, Venne et al., 2009), recent efficacy studies of ‘Foxy-2’ in Kenya showed no effective biocontrol ability of ‘Foxy-2’ against *S. hermonthica* (Avedi et al., 2014). These contradictory results were explained by potential genetic distinctions between Eastern and Western African *S. hermonthica* varieties, but also by abiotic and biotic environmental factors influencing the proliferation and hence efficacy of ‘Foxy-2’ in foreign ecosystems. Zimmermann et al. (2015), using a Fos specific and quantitative monitoring tool, followed the fate of BCA Fos after inoculation into foreign soil ecosystems, and showed that Fos proliferation was controlled by physico-chemical soil characteristics and by the availability of organic resources, for which Fos is in competition with indigenous microorganisms in the rhizosphere of the host crop. The latter fact requires particular attention as Fos is a soil borne fungus and proliferates saprotrophically and endophytically in crop rhizospheres and roots, respectively (Ndambi et al., 2011).

Soil microorganisms maintain critical soil functions including nutrient cycling as well as turnover and stabilization of soil organic matter (van der Heijden et al., 2008, Kunlanit et al., 2014). A range of soil microorganisms have been shown to suppress soil-borne plant diseases and to promote plant growth (Compant et al., 2005, Rasche et al., 2006a, Rasche et al., 2006b, Liu et al., 2007). With respect to resource acquisition in soils, it was recently speculated that there might exist a potential resource competition between Fos and indigenous soil microorganisms (Zimmermann et al., 2015). Hence, it could be hypothesized that the release of Fos in soils may have a considerable effect on the abundance and community composition of functionally relevant indigenous soil microorganisms which may in turn influence crop health and yield. The impact of the Fos strain ‘Foxy-2’ on the abundance of total indigenous bacterial communities and plant-beneficial prokaryotic nitrifiers in a maize rhizosphere was emphasized by Musyoki et al. (2015) who detected no negative side effects of ‘Foxy-2’ on root-associated bacteria.

In the study we present here, we focused on community dynamics of rhizosphere fungi as these may colonize similar niches as Fos and thus compete for similar resources in the rhizosphere (Winding et al., 2004). We put major emphasis on functionally relevant members of the fungal community focusing primarily on arbuscular mycorrhizal fungi (AMF) colonizing crop roots. The focus on AMF is justified due to their beneficial effects on crop growth and crop stress compensation (Smith and Smith, 2012). We studied the response of fungal communities to Fos inoculation in two contrasting (clayey Humic Nitisol versus sandy Ferric Alisol) soils from Kenya which were not naturally infested with Fos. A rhizobox experiment was conducted in which the selected soils were treated with the Fos strain ‘Foxy-2’ via seed coating of a tropical maize variety used as a test crop. Two additional factors were considered: (1) presence of *S. hermonthica*, and (2) application of *Tithonia diversifolia* residues, a widely used green manure in Sub-Saharan Africa (Gachengo et al., 1998; Jama et al., 2000, Opala et al., 2015), to cover the hypothesized resource competition effects. *T. diversifolia* is classified as high quality organic fertilizer with low C/N ratio (Chivenge et al., 2009) and provides an easily accessible C source and high N availability to stimulate indigenous fungal communities (Zimmermann et al., 2015). The response of the total fungal abundance was monitored at 14, 28 and 42 d after inoculation (DAI) using DNA-based quantitative polymerase chain reaction (qPCR), while fungal community composition (terminal restriction fragment length polymorphism (TRFLP) fingerprinting) and AMF taxa abundance (qPCR) were monitored at 42 DAI.

## 2 Material and methods

### 2.1 Rhizobox experiment

#### 2.1.1 Preparatory work

The model Fos isolate ‘Foxy-2’ was obtained from *S. hermonthica* collected from North Ghana (Abbasher et al., 1995). Taxonomic identification of the isolate was confirmed by Julius-Kühn-Institut (JKI), Berlin, Germany, where it is deposited under accession number BBA-67547-Ghana. Maize (*Z. mays* variety ‘WH507’, provided by Western Seed Company Ltd., Kitale, Kenya) was used as a test crop. The selected variety is highly preferred by smallholder farmers in Western Kenya due to its tolerance to *S. hermonthica*. Maize seeds were coated with dried ‘Foxy-2’ chlamydospore inoculum (1.15 × 10^5^ colony forming units per seed) homogenized into 20% arabic gum used as adhesive through a special seed treatment technology (Elzein et al., 2006; seed coating processed by SUET GmbH, Eschwege, Germany) to provide uniform inoculum coverage. *S. hermonthica* seeds (originating from Sudan) were surface sterilized according to Elzein et al. (2010) and germination viability of seeds (75%) was checked as described by Kroschel (2002).

#### 2.1.2 Rhizobox set-up

Rhizoboxes (3 × 7 × 20 cm) were filled with dry soils (165 g) derived from two contrasting field sites in the central highlands of Kenya: Embu (0° 30′ S, 37° 30′ E; 1380 m above sea level (a.s.l.)) and Machanga (0° 47′ S, 37° 40′ E; 1022 m a.s.l.). Soils differed greatly in physical properties: the Embu soil was a clayey Humic Nitisol (17% sand, 18% silt, 65% clay) derived from basic volcanic rocks, while the Machanga soil was a sandy Ferric Alisol (66% sand, 11% silt, 22% clay) derived from granitic gneisses (IUSS Working Group WRB, 2015). Each rhizobox was filled at the bottom with a 1 cm ground layer of vermiculite (grain size 3–8 mm) for drainage improvement. On top of this layer, soil adjusted to 50% water holding capacity was added.

Both soils were infected artificially with disinfected *S. hermonthica* seeds (20 mg seeds 165 g dry soil^−1^). *S. hermonthica* seeds were thoroughly mixed with the moist soils and pre-conditioned at 28 °C in the dark for 7 d (Kroschel, 2002). After this step, pre-germinated maize seedlings were introduced into the rhizoboxes. After planting of seedlings, a 1 cm layer of vermiculite was placed as the top layer to reduce evaporation.

Boxes were placed in an incubation chamber (12 h with artificial light (1000 μmol m^−2^ s^−1^) and 12 h darkness at 28/21 °C (day/night) for 6 weeks). Two and 4 weeks after the start of incubation, soil was fertilized with inorganic liquid fertilizer (4 ml each rhizobox with 0.2% Wuxal N-P-K (8-8-6), Aglukon GmbH, Düsseldorf, Germany) to avoid nutrient deficiency. In addition, a treatment with organic residues was included by incorporating air-dried and ground (particle size 1–3 mm) leaf and stem material of *T. diversifolia* (1 g dry matter 100 g dry soil^−1^) into soils before planting of maize seedlings. Non-fertilized treatments were included as controls.

The rhizobox experiment was arranged as a completely randomized design with 6 treatments with 3 replicates for each soil type: (i) uncoated maize seeds with no *S. hermonthica* (C), (ii) uncoated maize seeds and *S. hermonthica* (C + S), (iii) coated maize seeds with ‘Foxy-2’ (F), (iv) coated maize seeds with ‘Foxy-2’ and *S. hermonthica* (F + S), (v) coated maize seeds with ‘Foxy-2’ and *T. diversifolia* (F + T), and (vi) coated maize seeds with ‘Foxy-2’, *S. hermonthica* and *T. diversifolia* (F + S + T).

#### 2.1.3 Rhizosphere and bulk soil samplings

Rhizosphere samples for molecular analyses were taken 14, 28 and 42 d after inoculation (DAI). For this step, the rhizobox was opened and approximately 2 g of root adhered soil was taken carefully from several positions in order not to damage the root system. Rhizosphere soil was gently scraped off with sterile forceps and transferred into sterile sampling bags. Soil samples (bulk soil) for chemical analyses were obtained at 42 DAI. Rhizosphere soil samples were freeze dried and stored at −20 °C until molecular analysis, while bulk soils for chemical analyses were directly maintained at −20 °C. One proportion of the obtained rhizosphere soil samples was used to study the impact of ‘Foxy-2’ on indigenous prokaryotic communities (Musyoki et al., 2015) while another was used in the present study to assess the impact of ‘Foxy-2’ on indigenous fungal communities.

### 2.2 Analysis of fungal communities

#### 2.2.1 DNA extraction from rhizosphere samples

Total genomic DNA from rhizosphere samples was extracted using the Fast DNA^®^ Spin Kit for Soil (MP Biomedicals, Solon, OH, USA) following the manufacturer's instructions with slight modifications. Briefly, 0.4 g freeze-dried soil was bead-beated for 30 s with a beating power of 5.5 m s^−1^ using a FastPrep^®^-24 Instrument (MP Biomedicals). Concentration and quality of DNA were determined on a Nanodrop ND-1000 (Nanodrop Technologies, Wilmington, DE, USA) and DNA was stored at −20 °C.

A soil spiking experiment was conducted including the two soils used in the rhizobox experiment to account for soil type depending DNA extraction efficiencies influencing fungal gene copy recovery (Zimmermann et al., 2015). Briefly, 400 mg of freeze dried soil samples obtained from control sets of the rhizobox experiment were transferred into the beat beating tubes of the DNA extraction kit (MP Biomedicals). Soil samples in tubes were spiked with cloned ‘Foxy-2’ amplicons of known concentration (10^3^ ‘Foxy-2’ gene copies). Recovery of ‘Foxy-2’ amplicons after DNA extraction was determined using the qPCR protocol with ‘Foxy-2’ specific oligonucleotides Kb1::Kb2 as described in Zimmermann et al. (2015). Results of the soil spiking experiment verified that DNA extraction efficiency was soil type independent (Zimmermann et al., 2015).

#### 2.2.2 Total fungal abundance

Quantification of 18S rDNA gene copy numbers in soils was performed using oligonucleotides FF390 (5′-CGATAACGAACGAGACCT-3′) and FR1 (5′-AICCATTCAATCGGTAITCATTCA-3′) (Vainio and Hantula, 2000). Each reaction (20 μl) contained 5 ng DNA template, 10 μl of Power SYBR^®^ Green Master Mix (Applied Biosystems, Foster City, CA, USA), 0.2 μl T4 gene 32 protein (500 ng μl^−1^, MP Biomedicals), and 0.4 μM of each oligonucleotide. A cloned amplicon was used as standard in 10-fold serial dilutions of known DNA concentration (Kamolmanit et al., 2013). PCR runs were performed on a StepOnePlus™ Real-Time PCR System (Applied Biosystems). Cycling started with initial denaturation at 95 °C for 10 min, followed by 45 cycles of denaturation at 94 °C for 30 s, annealing at 50 °C for 30 s and polymerization at 70 °C for 1 min. Each DNA sample was processed in triplicate reactions, while standards were run in duplicates. Melting curve analysis of amplicons was conducted to confirm that fluorescence signals originated from specific amplicons and not from oligonucleotide dimers or other artifacts. An average reaction efficiency of 86% was achieved with R^2^ values consistently >0.98. Quantification of gene copies was calculated by comparing the values of threshold cycles (Ct) to the values of the crossing points of the linear regression line of the standard curve using StepOne™ software version 2.2 (Applied Biosystems).

It needs to be considered that the inoculated Fos strain ‘Foxy-2’ is part of the total fungal abundance. Both were quantified with the approach used and hence, it was likely that the abundance of ‘Foxy-2’ has enhanced the abundance of the indigenous fungal population. We have subtracted ‘Foxy-2’ abundance from the total fungal abundance using the following procedure. ‘Foxy-2’ was propagated in 5 ml potato dextrose broth at 28 °C for 3 d, followed by DNA extraction (UltraClean Microbial DNA Isolation Kit, MO BIO Laboratories Inc., Carlsbad, CA). Concentration and quality of ‘Foxy-2’ DNA were determined as described above. Five ng of ‘Foxy-2’ DNA was used as template #1 for Fos-specific qPCR (using oligonucleotides Kb1::Kb2 with the protocol published in Zimmermann et al. (2015) and template #2 for 18S rDNA qPCR (see above). The 5 ng ‘Foxy-2’ DNA template used for both qPCR assays corresponded to 2.3 × 105 ‘Foxy-2’ gene copies and 4.6 × 105 18S rDNA gene copies resulting in a ratio of 1:2 between ‘Foxy-2’ and 18S rDNA gene copies. Accordingly, the previously measured ‘Foxy-2’ gene copy numbers in the soils of the identical rhizobox experiment (Zimmermann et al., 2015) were first multiplied with factor 2 and then subtracted from total 18S rDNA gene copy numbers. This calculation resulted in the adjusted 18S rDNA gene copy numbers reflecting the abundance of the total indigenous fungal population.

#### 2.2.3 Fungal community composition

The fungal community composition was studied by terminal restriction fragment length polymorphism (TRFLP) analysis using the same oligonucleotide set as applied for 18S rDNA qPCR (Vainio and Hantula, 2000, Kamolmanit et al., 2013). The 18S rDNA gene was amplified in 25 μl reactions containing 5 ng DNA template, 1 × PCR buffer, 2 U Taq DNA polymerase (Bioline GmbH, Luckenwalde, Germany), 0.2 mM of each deoxynucleoside triphosphate (dNTP), 0.4 μM of each oligonucleotide (FF390::FR1), and 1.5 mM MgCl_2_. The forward oligonucleotide FF390 was labelled with the fluorescent dye FAM-6. PCRs were started with initial denaturation at 95 °C for 1 min, followed by 30 cycles consisting of a denaturation at 95 °C for 30 s, an annealing step at 52 °C for 45 s, and elongation at 72 °C for 2 min. Reactions were completed with a final elongation step at 72 °C for 10 min. Amplicons were purified using the Invisorb Fragment CleanUp Kit (Stratec Biomedical AG, Birkenfeld, Germany) following the manufacturer's instructions. For digestion, 200 ng of amplicons were incubated with 5 U *Msp*I restriction endonuclease (Promega GmbH, Mannheim, Germany) at 37 °C for 4 h followed by 65 °C for 20 min enzyme inactivation. Digested products were desalted with Sephadex™ G-50 (GE Healthcare) (Rasche et al., 2006a, Rasche et al., 2006b) and amended with 7.75 μl Hi-Di formamide (Applied Biosystems) and 0.25 μl internal size standard GeneScan™-500 ROX™ (Applied Biosystems). Mixtures were denaturated at 95 °C for 2 min, followed by immediate chilling on ice. TRFLP profiles were recorded on an ABI Genetic Analyzer 3130 (Applied Biosystems). Peak Scanner software (version 1.0, Applied Biosystems) was used to compare relative lengths of terminal-restriction fragments (T-RFs) with the internal size standard and to compile electropherograms into numeric data sets, in which T-RF length and height >100 fluorescence units (Fredriksson et al., 2014) were used for statistical profile comparison. TRFLP profiles used for statistical analyses were normalized according to Dunbar et al. (2000). A requirement for analyzing ‘Foxy-2’ induced alterations in indigenous fungal community composition was the deletion of ‘Foxy-2’ T-RF from TRFLP profiles. To account for this, we used the following procedure: Fos strain ‘Foxy-2’ was propagated in 5 ml potato dextrose broth at 28 °C for 3 d, followed by DNA extraction (UltraClean Microbial DNA Isolation Kit, MO BIO Laboratories Inc., Carlsbad, CA). Concentration and quality of ‘Foxy-2’ DNA were determined as described above. Five ng of ‘Foxy-2’ DNA was used as template for 18S rDNA PCR with oligonucleotides described above in triplicate reactions. PCR amplicons were purified using the Invisorb^®^ Fragment CleanUp (Stratec Molecular GmbH, Berlin, Germany), quantified as described above and adjusted to the recommended DNA concentration for sequencing. Sequencing was done with the 18S rDNA primer FR1 (LGC Genomics GmbH, Berlin, Germany) and 18S rDNA sequences of ‘Foxy-2’ were submitted to http://www.restrictionmapper.org/to identify restriction cutting sites with the enzyme *Msp*I used for TRFLP analysis. The resulting T-RF of ‘Foxy-2’ with 168 base pair length was deleted from TRFLP profiles.

#### 2.2.4 AMF taxa abundance

AMF taxon-specific oligonucleotides specifically developed for qPCR assays (Thonar et al., 2012) were used in this study since universal AMF primers (i.e., NS31::AM1, AML1::AML2 and NS31::AML2; Helgason et al., 1998, Lee et al., 2008, Simon et al., 1992) lack in specificity for the intended Sybr Green qPCR approach (Kohout et al., 2014). Moreover, it is worthwhile noting that these universal AMF oligonucleotides were not appropriate for qPCR assays as they produce amplicon lengths up to 1800 base pairs. Hence, monitored AMF taxa in this study served as model organisms to investigate potential non-target effects of the BCA ‘Foxy-2’ on indigenous AMF. Thonar et al. (2012) developed qPCR oligonucleotides for five major AMF taxa (i.e., *Rhizoglomus irregulare*, *Funneliformis mosseae*, *Gigaspora margarita*, *Cetraspora pellucida*, *Claroideoglomus claroideum)*. In a first step, the two soils used in the rhizobox experiment (i.e., sandy and clayey soil) were checked for any occurrence of the five AMF taxa. For this, we used a nested PCR approach to obtain a higher sensitivity. PCR #1 with oligonucleotides LR1::FLR2 (Jansa et al., 2002) was amplified in 25 μl reactions containing 10 ng soil DNA template, 1 × PCR buffer, 2 U ACCUZYME DNA polymerase (Bioline GmbH, Luckenwalde, Germany), 0.2 mM of each dNTP, 0.4 μM of each oligonucleotide (LR1::FLR2), and 1.5 mM MgCl_2_ with cycling conditions described in Jansa et al. (2002). Amplicons of PCR #1 were diluted 1:200 and used as template (2 μl) for PCR #2 with taxon-specific AMF oligonucleotides developed by Thonar et al. (2012) using 25 μl reactions containing 1 × PCR buffer, 2 U ACCUZYME DNA polymerase (Bioline GmbH, Luckenwalde, Germany), 0.2 mM of each dNTP, 0.4 μM of each oligonucleotide, and 2.0 mM MgCl_2_. PCR #2 was started with initial denaturation at 95 °C for 5 min, followed by 35 cycles consisting of a denaturation at 95 °C for 1 min, an annealing step (Table 1) for 45 s, and elongation at 72 °C for 2 min. Reactions were completed with a final elongation step at 72 °C for 10 min. PCR #2 amplicons were visualized with GelRed™ (Biotrend Chemikalien GmbH, Cologne, Germany) staining in an agarose gel (1% agarose (Carl Roth GmbH, Karlsruhe, Germany)) following electrophoresis (120 V, 45 min). The nested PCR approach identified two AMF taxa naturally occurring in each soil used in our rhizobox experiment (i.e., *C. pellucida* and *G. margarita* in the clayey soil and *C. pellucida* and *C. claroideum* in the sandy soil).

The qPCR assays were conducted for the identified AMF taxa as follows: Each qPCR (20 μl) contained 50 ng DNA template, 10 μl of Brilliant III Ultra-Fast SYBR^®^Green QPCR Master Mix (Agilent Technologies, Santa Clara, USA), 0.3 μl of 1:50 diluted passive reference dye (Agilent Technologies), 0.2 μl T4 gene 32 protein (500 ng μl^−1^, MP Biomedicals) and 0.5 μM of each oligonucleotide corresponding to the assayed AMF taxa. A cloned amplicon was used for each AMF taxa as standard in 10-fold serial dilutions of known DNA concentration. PCR runs were performed on a StepOnePlus™ Real-Time PCR System (Applied Biosystems). Cycling started with initial denaturation at 95 °C for 10 min, followed by 40 cycles of denaturation at 94 °C for 15 s, individual annealing temperature (Table 1) for 30 s and polymerization at 72 °C for 1 min. An additional step at 76 °C for 30 s was included for signal detection. Occasionally, small peaks occurred in the melting curve between 72 and 75 °C due to oligonucleotide dimers not detected by electrophoresis in a 1.5% agarose gel (data not shown). To avoid measurement of fluorescence signal emitted by these oligonucleotide dimers, fluorescence of target amplicon (*C. pellucida* amplicon T_m_ = 81 °C, *G. margarita* amplicon T_m_ = 82 °C, *C. claroideum* amplicon T_m_ = 80 °C) was detected at 76 °C. Each DNA sample was processed in triplicate reactions, while standards were run in duplicates. Melting curve analysis of amplicons was conducted to confirm reaction quality as described above. Quantification of gene copies was processed as described above and average qPCR reaction efficiencies were 89% for *C. pellucida*, 86% for *G. margarita*, and 84% for *C. claroideum* with R^2^ values consistently >0.98.

### 2.3 Measurement of soil chemical parameters

For statistical purposes, data on total carbon (TC), total nitrogen (N_t_), extractable organic C (EOC), total extractable N (TEN) and pH of soils was retrieved from Musyoki et al. (2015). Plant-available phosphorus (P_av_) was extracted with the Bray-Kurtz P1 test (Bray and Kurtz, 1945) and content of P_av_ in extracts was quantified at 882 nm on a spectrophotometer (SPECORD 50, Analytik Jena AG).

### 2.4 Statistical analyses

Each rhizobox was sampled at 3 sequential dates (DAI 14, 28 and 42). Therefore, a repeated measures analysis with an autoregressive covariance structure using the ‘nlme’ package (Pinheiro et al., 2014) combined with post hoc Tukey-B tests using the ‘lsmeans’ package (Lenth, 2013) in R (R Core Team, 2013) was performed to determine effects of the 5 factors ‘Foxy-2’, ‘*S. hermonthica*’, ‘*T. diversifolia*’, ‘Soil type’ and ‘Sampling date’ on 18S rDNA abundance. AMF species abundance was monitored only at 42 DAI. Hence, for analysis of AMF species abundance, a multifactorial ANOVA was applied in R combined with post hoc Tukey-B tests with the factors mentioned above, but excluding factor ‘sampling date’. Pearson's correlation coefficients were used to assess significant relations between total fungal and AMF species abundance and soil chemical parameters (Musyoki et al., 2015) across all treatments in each soil at 42 DAI (6 observations).

TRFLP data sets were analysed using Bray-Curtis similarity coefficients (Legendre and Legendre, 1998). A similarity matrix was generated for all possible pairs of samples for each target gene. This similarity matrix was used for analysis of similarity (ANOSIM) statistics (Clarke, 1993) to test if the composition of target fungal communities was altered by factors ‘Foxy-2’, ‘*S. hermonthica*’, ‘*T. diversifolia*’ and ‘Soil type’. ANOSIM is based on rank similarities between the sample matrix and produces a test statistic ‘R’ (Rees et al., 2005). A ‘global’ R was first calculated in ANOSIM, which evaluated the overall effect of a factor in the data set. This step was followed by a pair wise comparison, whereby the magnitude of R indicated the degree of separation between two tested communities. An R score of 1 indicated a complete separation, while 0 indicated no separation (Rees et al., 2005). Treatment separation was visualized by non-metric multidimensional scaling (nMDS). nMDS calculates a stress value indicating the fitness of similarity ranking, where a stress value below 0.2 warrants a justified treatment separation (Clarke and Warwick, 2001). Calculation of similarity coefficients, ANOSIM and nMDS were carried out using Primer for Windows version 6 (Primer-E Ltd., Plymouth, UK). To verify if considered soil chemical parameters (Musyoki et al., 2015) were decisive for the observed treatment-driven community composition shifts of the total fungal population, the DistLM procedure of PERMANOVA+ in Primer v6 (Primer-E Ltd.) was used (Clarke and Gorley, 2006). This procedure calculates a linear regression between the diversity of fungal communities using the Shannon diversity index and log transformed soil chemical data (Legendre and Anderson, 1999).

## 3 Results

### 3.1 Total fungal abundance

**Fig. 1:**
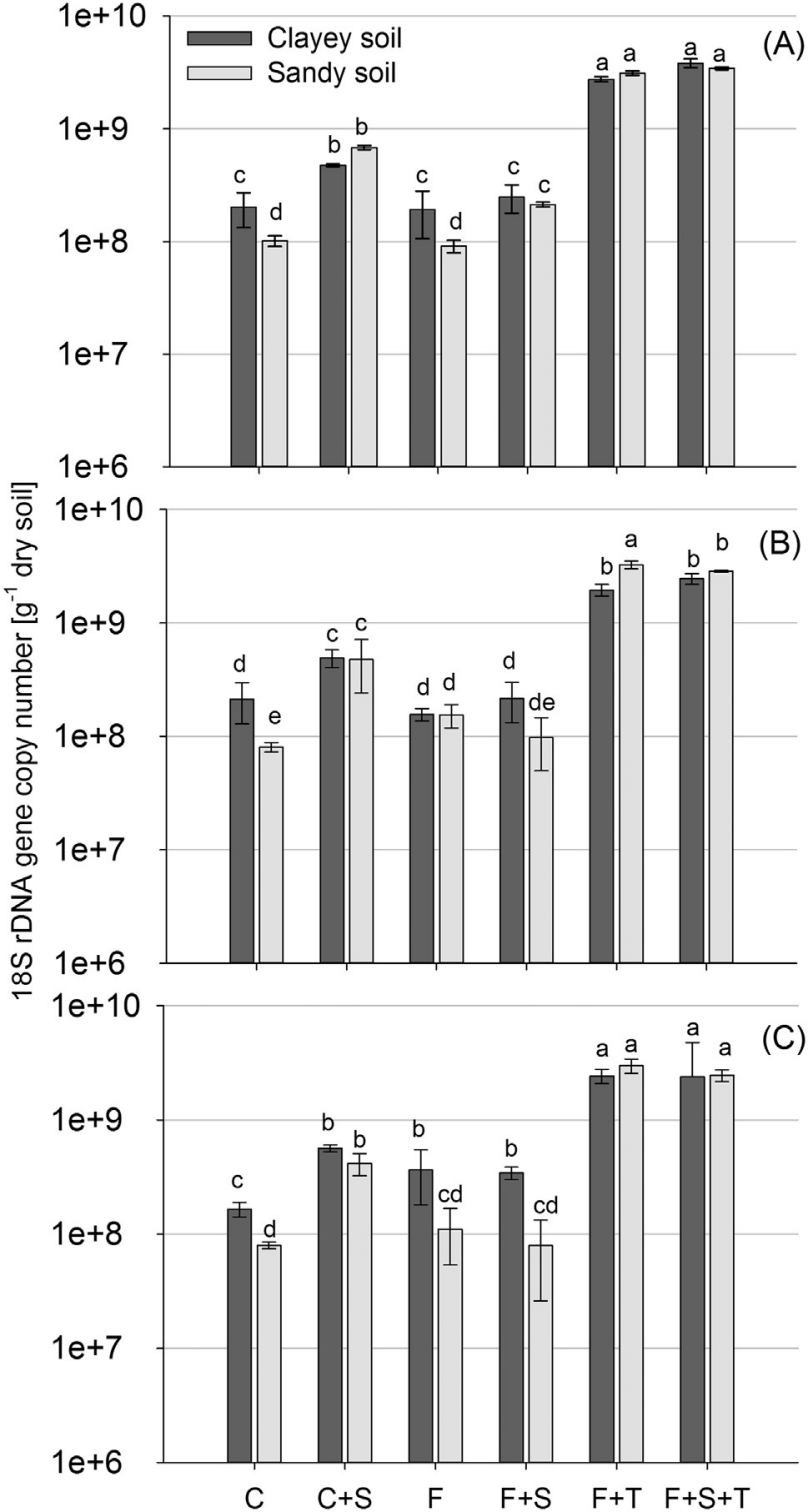
Adjusted total fungal abundance based on 18S rDNA gene copy numbers determined at 14 (A), 28 (B) and 42 (C) after ‘Foxy-2’ inoculation in the two soils (‘clayey’ (Humic Nitisol), ‘sandy’ (Ferric Alisol)). Different letters indicate significant differences at *P* < 0.05 and error bars represent standard error. Treatment codes are: uncoated maize (C), uncoated maize with *S. hermonthica* (C + S), coated maize with ‘Foxy-2’ (F), coated maize with ‘Foxy-2’ and *S. hermonthica* (F + S), coated maize with ‘Foxy-2’ and *T. diversifolia* (F + T), and coated maize with ‘Foxy-2’, *S. hermonthica* and *T. diversifolia* (F + S + T).

Treatment ‘Foxy-2’ (F) promoted the total fungal abundance (18S rDNA gene copies) at 28 DAI in the sandy soil (*P* < 0.01) and at 42 DAI in the clayey soil (*P* < 0.001) compared to the control treatment (C) (Fig. 1). No *S. hermonthica* root attachment and emergence was detected within the 42 d incubation. Nonetheless, treatment ‘*S. hermonthica*’ (C + S) induced a stimulating effect on total fungal abundance throughout all sampling dates compared to treatment C (*P* < 0.001). The stimulating effect of *S. hermonthica* on total fungal abundance was less pronounced when inoculated together with ‘Foxy-2’ (F + S) (*P* < 0.01). Addition of *T. diversifolia* residues (F + T, F + S + T) promoted total fungal abundance in both soils at all sampling dates compared to treatment F (*P* < 0.001).

### 3.2 Fungal community composition

In the clayey soil, ANOSIM of TRFLP profiles revealed the strongest community separation between control treatments (C, C + S) and *T. diversifolia* amended treatments (F + T, F + S + T) with R = 1 (Table 2). Control treatment (C) versus ‘Foxy-2’ treatment (F) resulted in R = 0.333. In the same soil, treatment C + S induced a community composition distinction with treatment C (R = 0.556). Moreover, treatment F was different from *T. diversifolia* amended treatment (F + T) (R = 0.915) and F + S + T (R = 0.989).

In the sandy soil, a clear community difference was detected between control treatment C versus *T. diversifolia* amended treatments (F + T, F + S + T) with R = 1. Treatment F showed a community distinction to the *T. diversifolia* amended treatments (F + T, F + S + T) with R = 1. Treatment C was only slightly different from treatment F (R = 0.259), while treatments C and C + S showed a community difference of R = 0.364.

**Fig. 2:**
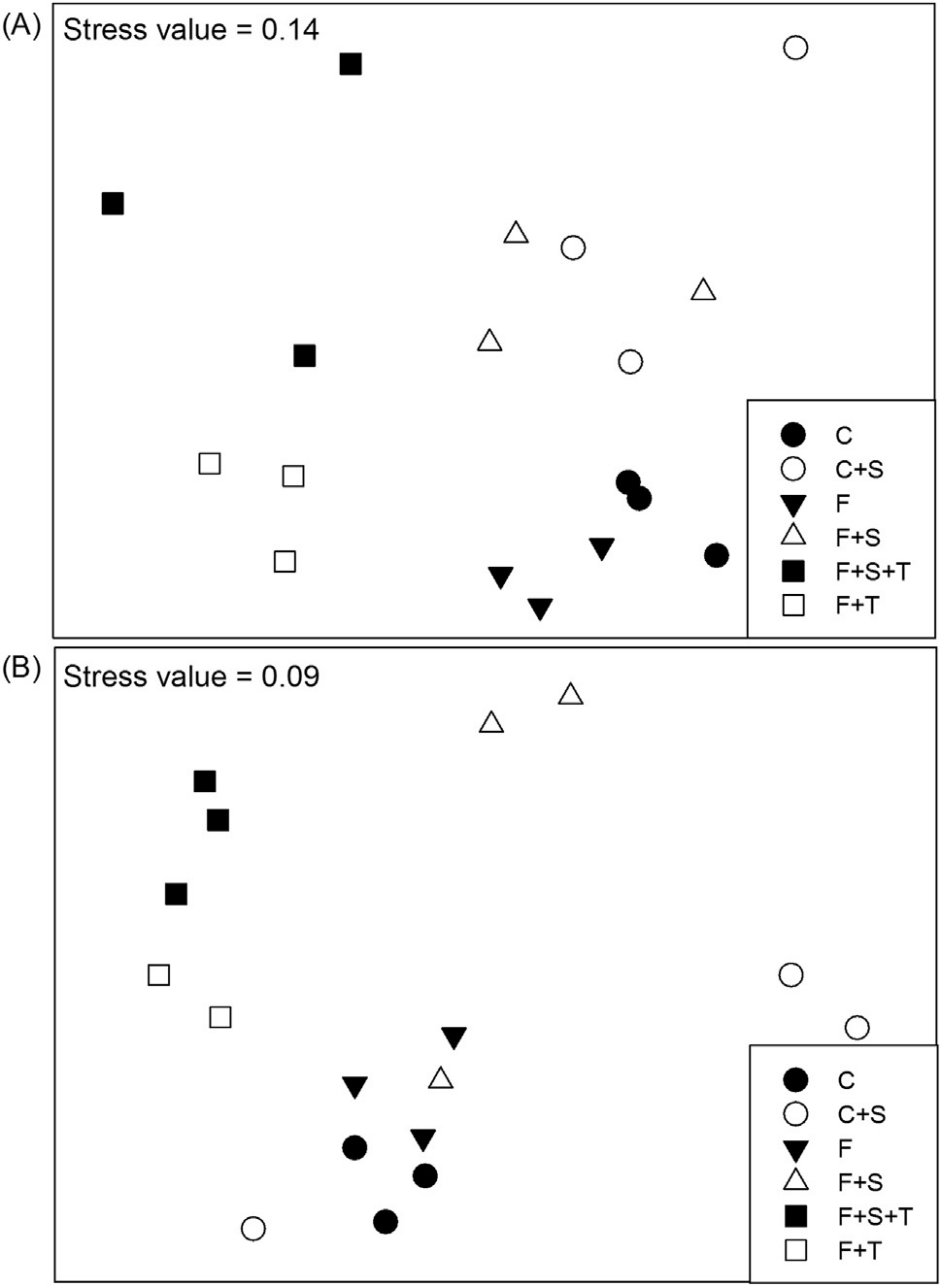
Bray-Curtis similarity-based non-metric multidimensional scaling plot (nMDS) of normalized TRFLP data obtained from *Msp*I-digested 18S rDNA amplicons visualizing the differences in fungal community composition in the clayey (A) and sandy (B) soil according to the following treatments: uncoated maize (C), uncoated maize with *S. hermonthica* (C + S), coated maize with ‘Foxy-2’ (F), coated maize with ‘Foxy-2’ and *S. hermonthica* (F + S), coated maize with ‘Foxy-2’ and *T. diversifolia* (F + T) and coated maize with ‘Foxy-2’, *S. hermonthica* and *T. diversifolia* (F + S + T).

Effects of factors ‘Foxy-2’, ‘*S. hermonthica*’ and ‘*T. diversifolia*’ on the community composition of the total fungal population were confirmed by nMDS showing clear separations between treatments with stress values of 0.14 in the clayey (Fig. 2A) and 0.09 in the sandy (Fig. 2B) soils.

### 3.3 AMF taxa abundance

**Fig. 3:**
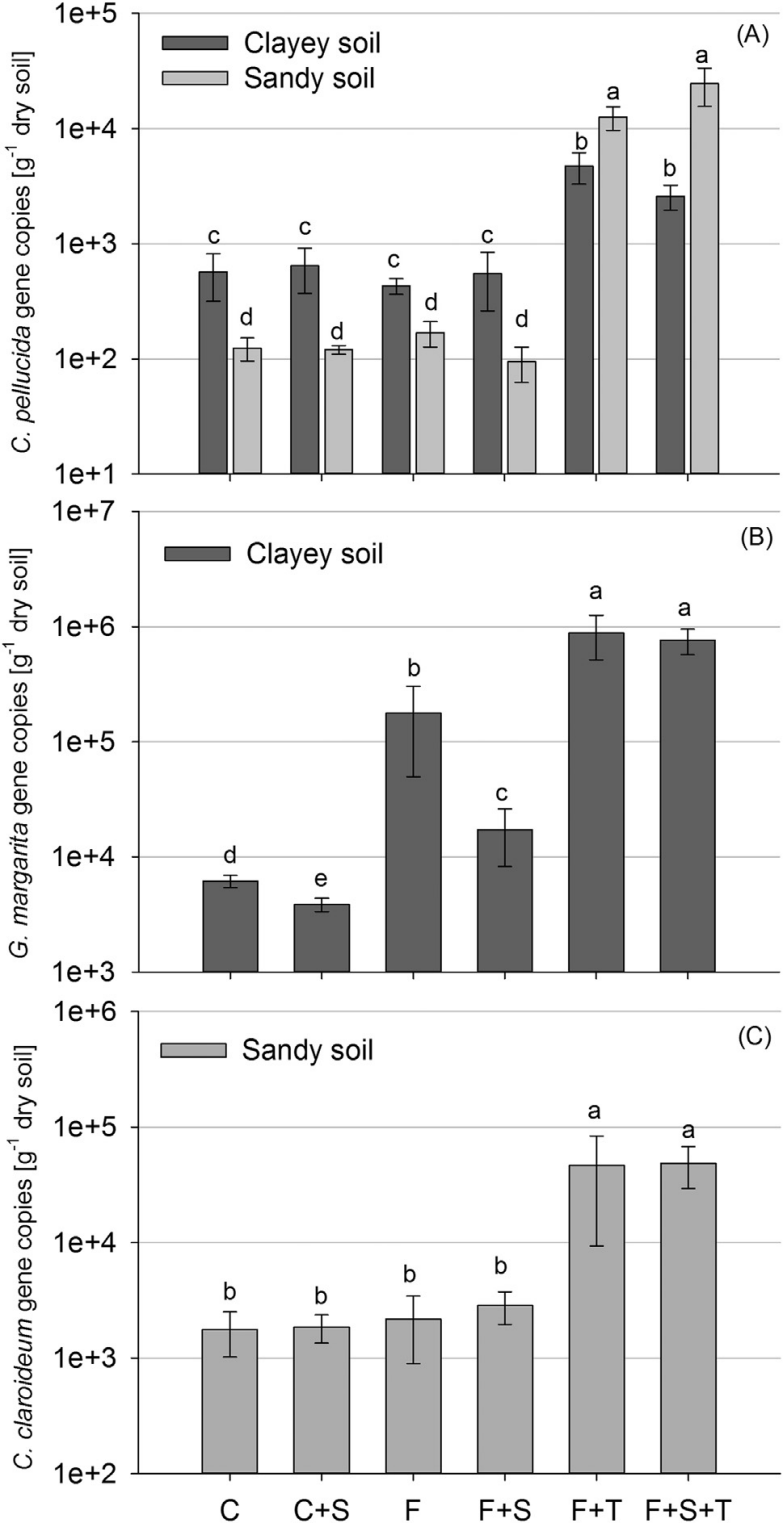
AMF taxa abundance at 42 DAI in the two soils (‘clayey’ (Humic Nitisol), ‘sandy’ (Ferric Alisol)). Different letters indicate significant differences at *P* < 0.05 and error bars represent standard error. Treatment codes are: uncoated maize (C), uncoated maize with *S. hermonthica* (C + S), coated maize with ‘Foxy-2’ (F), coated maize with ‘Foxy-2’ and *S. hermonthica* (F + S), coated maize with ‘Foxy-2’ and *T. diversifolia* (F + T), and coated maize with ‘Foxy-2’, *S. hermonthica* and *T. diversifolia* (F + S + T).

AMF *C. pellucida* was detected in both soil types, while *G. margarita* and *C. claroideum* were detected only in the clayey and sandy soils, respectively (Fig. 3). Abundance of *C. pellucida* was higher in the clayey than the sandy soil when not treated with *T. diversifolia* residues (*P* < 0.001). An opposite effect was detected for *T. diversifolia* residue treatments (*P* < 0.001). The highest *G. margarita* abundance was detected in the *T. diversifolia* treatments (F + T, F + S + T) (*P* < 0.001). Additionally, its abundance was promoted by ‘Foxy-2’ (F) compared to the control (C) (*P* < 0.001). Conversely, a suppressive effect on *G. margarita* abundance was detected under *S. hermonthica* treatments (C + S, F + S) compared to the respective controls (C, F). *C. claroideum* abundance was promoted in *T. diversifolia* treatments compared to all other treatments (*P* < 0.001).

### 3.4 Correlation of community abundance and composition with soil chemical data

**Fig. 4:**
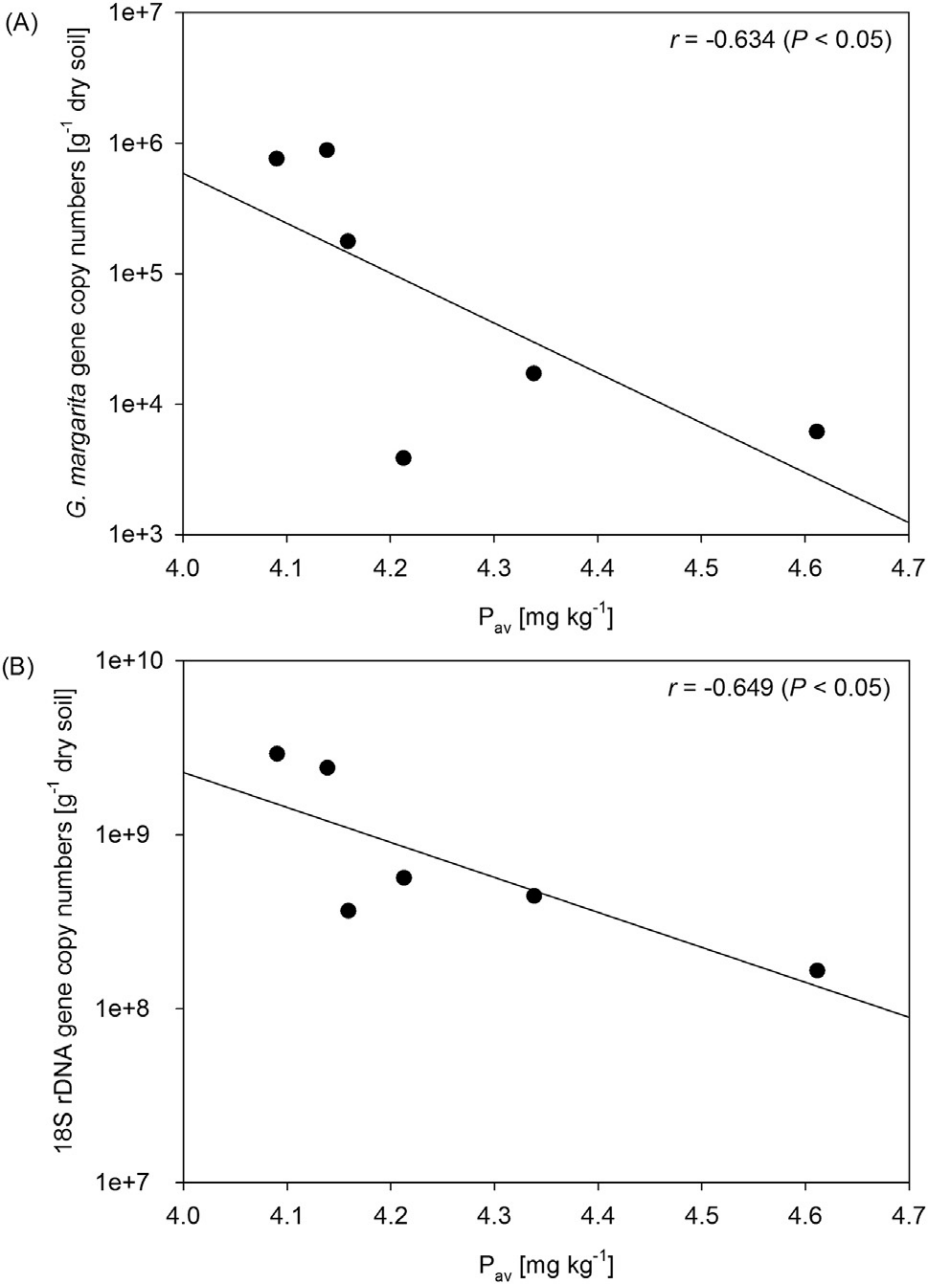
Relationship between plant-available phosphorus (P_av_) content and *G. margarita* (A) and 18S rDNA (B) gene copy numbers. Pearson's linear correlation coefficients (*r*) and *P* values are given in each plot.

For total fungal abundance, 18S rDNA gene copy numbers showed in the clayey soil a negative correlation with plant-available P (P_av_) (*r* = −0.649, *P* < 0.05, Fig. 4B, Table 3). *C. pellucida* abundance revealed in the sandy soil positive correlations with extractable organic nitrogen (EON) (*r* = 0.563, *P* < 0.05), while *G. margarita* abundance in the clayey soil was negatively correlated with P_av_ (*r* = −0.634, *P* < 0.05, Fig. 4A, Table 3). Moreover, there was a positive correlation in the sandy soil between Fos gene copy numbers (Zimmermann et al., 2015) and adjusted 18S rDNA gene copy numbers (*r* = 0.741, *P* < 0.01).

Shannon diversity indexes calculated from the TRFLP data of total fungal communities and log transformed soil chemical data revealed in the clayey soil positive correlations for soil pH (*r* = 0.775, *P* < 0.001), EOC (*r* = 0.748, *P* < 0.001), NH_4_^+^ (*r* = 0.606, *P* < 0.01), TC (*r* = 0.602, *P* < 0.01) and P_av_ (*r* = 0.551, *P* < 0.05). In the sandy soil, positive correlations were detected for soil pH (*r* = 0.669, *P* < 0.01), N_t_ (*r* = 0.645, *P* < 0.01), EON (*r* = 0.640, *P* < 0.01), EOC (*r* = 0.621, *P* < 0.01), TC (*r* = 0.599, *P* < 0.01) and NH_4_^+^ (*r* = 0.497, *P* < 0.05).

## 4 Discussion

### 4.1 Impact of ‘Foxy-2’ on indigenous AMF

In the present study, we assayed the potential impacts of the BCA ‘Foxy-2’ on the total indigenous soil fungal community, as well as fungal community parts with proven beneficial functions (i.e., AMF), colonizing the rhizosphere of maize. One major finding was the promoting effect of ‘Foxy-2’ on the abundance of AMF *G. margarita*, while the other two monitored AMF taxa remained unaffected. Similarly, *G. margarita* abundance was suppressed by *S. hermonthica* which was compensated when ‘Foxy-2’ was inoculated. Hence, our findings implied a tripartite interaction between ‘Foxy-2’, AMF *G. margarita* and *S. hermonthica*. The likely linkage between *S. hermonthica* and AMF was the root exudate ‘strigolactone’, a known stimulant of *S. hermonthica* germination (Yoneyama et al., 2010) and also AMF root colonization (Besserer et al., 2006, Bouwmeester et al., 2007). Exudation of strigolactones is specifically increased when crops are exposed to phosphorus (P) deficiency. Under such circumstances, the crop attracts symbiotic AMF to compensate for this limitation (Yoneyama et al., 2012, Czarnecki et al., 2013, Jamil et al., 2014). Consequently, we determined a negative correlation between plant available P and abundance of AMF *G. margarita* along with the total fungal community, a finding in line with earlier reports (Ryan et al., 2000, Smith and Read, 2008). Likewise, root colonization by AMF (i.e., *Glomus clarum*, *G. margarita*; Othira et al., 2012) was shown to reduce the infection of crops (e.g., sorghum, maize) by *S. hermonthica* (Gworgwor and Weber, 2003, Lendzemo et al., 2005, Lendzemo et al., 2007, Othira et al., 2012) due to down-regulated strigolactone formation following mycorrhizal colonization of crop roots (Lendzemo et al., 2009, López-Ráez et al., 2011, Aroca et al., 2013).

In contrast to earlier studies (Gworgwor and Weber, 2003, Lendzemo et al., 2005, Lendzemo et al., 2007, Othira et al., 2012), where AMF presence suppressed *S. hermonthica*, our results showed for the first time a suppressive effect of *S. hermonthica* on AMF *G. margarita.* Which actual mechanism underlies this observed interaction remains unclear, especially under the short experimental period during which no *S. hermonthica* root attachment or emergence was visually detected. Assuming that germination of *S. hermonthica* seeds started at the end of the experiment, competition for infection sites on the crop roots may have been the main driver of the interaction between *S. hermonthica* and AMF *G. margarita*. In this context, the role of cytotoxic and antifungal compounds (i.e., iridoids; da Silva et al., 2007, Céspedes et al., 2014) potentially secreted by *S. hermonthica* (Rank et al., 2004) may have been decisive.

Our results indicated that the promoting effect of ‘Foxy-2’ compensated for the suppressive effect of *S. hermonthica* on AMF *G. margarita* which obviously represented an additional benefit when implementing ‘Foxy-2’ as BCA. Several studies have reported enhanced mycorrhization of crop roots when AMF and saprotrophic *F. oxysporum* were applied simultaneously (Garcia-Romera et al., 1998, Fracchia et al., 2000, Diedhiou et al., 2003). The mechanism behind this obvious interaction is, however, yet to be understood.

Organic N fertilization (i.e., *T. diversifolia* residues) promoted the abundance of all studied AMF taxa which was corroborated by positive correlations with extractable organic nitrogen (EON) contents in soils as well as by earlier findings by Gryndler et al. (2005). Hodge and Fitter (2010) verified that AMF scavenged substantial amounts of nitrogen (N) from decomposing organic materials not only for the transfer to the plant during symbiosis, but also as an important N source for maintenance of their own metabolism. Furthermore, Aleklett and Wallander (2012) confirmed the ability of high quality fertilizers with low C/N ratio (i.e., *T. diversifolia*, Chivenge et al., 2009) to stimulate AMF abundance in contrast to low quality, ineffective fertilizers with high C/N ratio. Accordingly, this justified the consideration of *T. diversifolia* as organic fertilization treatment to compensate for potential suppressive effects of ‘Foxy-2’ on indigenous soil fungal communities, through providing additional N and C resources to the rhizosphere microbial community. Our results indicated, however, that *T. diversifolia* in conjunction with ‘Foxy-2’ was not essentially required since no suppressive effects of ‘Foxy-2’ on AMF were detected. On the other hand, as the promoting effect of *T. diversifolia* on AMF abundance was justified in this study, it may be further considered for general soil fertility improvement by resource-limited small-holder farmers.

### 4.2 Impact of ‘Foxy-2’ relative to other factors on total fungal community

We found a promoting effect of ‘Foxy-2’ on total fungal abundance in both soil types, although these were only transient and inconsistent. ‘Foxy-2’ induced higher total fungal abundance in the sandy soil at DAI 28 which ceased at DAI 42. In the clayey soil, the promoting effect of ‘Foxy-2’ on total fungal abundance was delayed and only visible at DAI 42, a finding similar to that of Gullino et al. (1995) and Ghini et al. (2000). Further, Karpouzas et al. (2011) detected only minor temporary effects of an endophytic *Fusarium* strain on the fungal community in the tomato rhizosphere of a sandy loam soil, while a tomato-pathogenic *Fusarium* strain caused long lasting effects on the respective fungal community.

*T. diversifolia* exhibited stronger effects on total fungal abundance than ‘Foxy-2’. This was in accordance with recent findings by Kamolmanit et al. (2013) and Lee et al. (2013) showing that higher N availability in organic residues increased total fungal abundance in soils in contrast to organic residues with low N availability or mineral fertilizer. Our results were further substantiated by Poll et al. (2010) and España et al. (2011) who confirmed that fast-growing opportunistic fungi were stimulated by easily accessible C sources and high N availability which corresponded to the *T. diversifolia* effect observed in our study. According to the organic input induced alterations of the fungal abundance, we found similar responses of the fungal community composition, as supported by positive correlations between Shannon diversity indexes with chemical soil properties (e.g., ammonia, N_t_, EON). These findings matched those of Yu et al. (2015) who suggested that the soil microbial community composition is mainly structured by physico-chemical soil characteristics including nutrient status.

## 5 Conclusions

The exclusion of non-target effects of introduced microbial BCAs on the indigenous soil microbial community is essential for the registration and commercialization of a BCA, such as ‘Foxy-2’. In the current study, we evaluated the effects of the fungal BCA ‘Foxy-2’ on the total indigenous soil fungal abundance and composition, as well as fungal community members with proven beneficial functions (i.e., AMF). A highlight of our study was the promoting effect of ‘Foxy-2’ on the AMF *G. margarita*, while the other two monitored AMF taxa (i.e., *C. pellucida*, *C. claroideum*) remained unaffected. Hence, no suppressive effects of ‘Foxy-2’ on these selected AMF are to be expected when implementing ‘Foxy-2’ in the field as an environmentally safe BCA.

Further research should emphasize the promoting effect of ‘Foxy-2’ on AMF *G. margarita* under long-term conditions, and consider a broader variety of crops (i.e., sorghum) since several AMF taxa show a high host specialization (Martínez-García and Pugnaire, 2011). In this context, additional AMF taxa need to be tested with respect to their compatibility with ‘Foxy-2’ emphasizing those AMF taxa with proven *S. hermonthica* suppression (i.e., *G. clarum*; Othira et al., 2012).

The present study was based on a short-term, controlled rhizobox experiment and, hence, similar experiments should be conducted under natural field conditions over a longer time period to gain a more detailed insight into the ecological effects of ‘Foxy-2’. These future experiments should account for relevant factors such as crop variety and development, a broader range of fertilization regimes and soil types, as well as seasonal characteristics including rainfall and temperature patterns.

## Figures and Tables

**Table 1:** Sequences of oligonucleotides (Thonar et al., 2012) and corresponding annealing temperatures used for quantitative PCR of the different AMF taxa.

Target AMF species	Oligonucleotide sequences (5′–3′)	Annealing temperatures (°C)
*Cetraspora pellucida*	AGAAACGTTTTTTACGTTCCGGGTTG	54
	CCAAACAACTCGACTCTTAGAAATCG	
*Gigaspora margarita*	CTTTGAAAAGAGAGTTAAATAG	48
	GTCCATAACCCAACACC	
*Claroideoglomus claroideum*	GCGAGTGAAGAGGGAAGAG	52
	TTGAAAGCGTATCGTAGATGAAC	

**Table 2:** Analysis of similarity (ANOSIM) of TRFLP datasets based on pair wise comparison of treatments. The magnitude of R indicates the degree of separation between two tested communities. An R score of 1 indicates a complete separation, while 0 indicates no separation.

Soil	Treatment (pair wise comparison)	R statistic	Significance level
Clayey soil (Embu)	C, C + S	0.556	0.01*
	C, F	0.333	0.04*
	C, F + S	0.593	0.01*
	C, F + T	1	0.01*
	C, F + S + T	1	0.01*
	C + S, F	0.333	0.04*
	C + S, F + S	0.111	0.06^ns^
	C + S, F + T	1	0.01*
	C + S, F + S + T	1	0.01*
	F, F + S	0.667	0.01*
	F, F + T	0.915	0.01*
	F, F + S + T	0.989	0.01*
	F + S, F + T	1	0.01*
	F + S, F + S + T	0.667	0.01*
	F + T, F + S + T	0.407	0.01*
Sandy soil (Machanga)	C, C + S	0.364	0.06^ns^
	C, F	0.259	0.06^ns^
	C, F + S	0.630	0.01*
	C, F + T	1	0.01*
	C, F + S + T	1	0.01*
	C + S, F	0.259	0.08^ns^
	C + S, F + S	0.222	0.06^ns^
	C + S, F + T	0.667	0.01*
	C + S, F + S + T	0.444	0.02*
	F, F + S	0.37	0.02*
	F, F + T	1	0.01*
	F, F + S + T	1	0.01*
	F + S, F + T	0.926	0.01*
	F + S, F + S + T	0.926	0.01*
	F + T, F + S + T	0.333	0.06^ns^

Significance levels: ns.: P > 0.05; *P < 0.05; **P < 0.01; ***P < 0.001.

Treatment codes: uncoated maize with no *S. hermonthica* (C), uncoated maize and *S. hermonthica* (C + S), coated maize with ‘Foxy-2’ (F) and coated maize with ‘Foxy-2’ and *S. hermonthica* (F + S), as well as coated maize with ‘Foxy-2’, *S. hermonthica* and *T. diversifolia* (F + S + T) and without *S. hermonthica* (F + T).

**Table 3:** Pearson's linear correlation coefficients between target gene abundance (18S rDNA, *C. pellucida*, *C. claroideum* and *G. margarita*) and soil chemical data at 42 DAI.

Soil	Target gene	TC [g kg^−1^]	N_t_ [g kg^−1^]	EOC [mg kg^−1^]	EON [mg kg^−1^]	NH_4_^+^ [mg kg^−1^]	NO_3_^−^ [mg kg^−1^]	P_av_ [mg kg^−1^]	Soil pH
Clayey soil (Embu)	18S rDNA	0.028^ns^	0.171^ns^	0.463^ns^	0.109^ns^	0.398^ns^	0.062^ns^	−0.649*	0.196^ns^
	*C. pellucida*	−0.187^ns^	0.065^ns^	0.268^ns^	−0.040^ns^	0.392^ns^	0.223^ns^	−0.409^ns^	0.080^ns^
	*G. margarita*	−0.089^ns^	0.127^ns^	0.252^ns^	0.031^ns^	0.420^ns^	0.180^ns^	−0.634*	0.093^ns^
Sandy soil (Machanga)	18S rDNA	0.121^ns^	0.154^ns^	0.199^ns^	0.223^ns^	0.121^ns^	0.066^ns^	−0.311^ns^	0.121^ns^
	*C. pellucida*	0.459^ns^	0.505^ns^	0.464^ns^	0.563*	0.257^ns^	0.043^ns^	0.069^ns^	0.404^ns^
	*C. claroideum*	0.215^ns^	0.283^ns^	0.243^ns^	0.307^ns^	0.159^ns^	−0.016^ns^	−0.103^ns^	0.198^ns^

Abbreviations: TC: Total carbon, Nt: Total nitrogen, EOC: Extractable organic carbon, EON: Extractable organic nitrogen, NH4+: ammonia, NO3−: nitrate, Pav: Plant-available phosphorus.

Significance levels: ns: *P* > 0.05; *P < 0.05; **P < 0.01; ***P < 0.001.
